# Cases of Fever, with Destructive Parotitis

**Published:** 1873-02-01

**Authors:** J. F. Kelsey

**Affiliations:** Peshtigo, Wis.


### CASES OF FEVER, WITH DESTRUCTIVE PAROTITIS.

BY J. F. KELSEY, M.D., OF PESHTIGO, WIS.

I have had during my practice two cases which at the time I considered of peculiar importance. Inasmuch as I never had seen any recorded cases like them, and believe that they are in no wise common, I will make the statement in general terms, hoping not only to elicit discussion but that it may be the means of throwing some light upon any which shall be at this time, or any future period, placed in the same position as I have been myself.

Case I.—In the spring of 1866, while practicing in Albert Lee, Minnesota, I was called to see J. E. Smith, then county treasurer of Freeborn county, who was laboring under a severe attack of bilious pneumonia, involving the liver and right lung only. It was of such severity that his recovery was considered doubtful until about the end of the first week of the disease. He then commenced to improve, but after three or four days the right parotid gland became very painful, swelled and inflamed, in consequense of the mumps setting in at this time. There was an epidemic of this particular malady in the neighborhood, and his own children—three of them—were having the disease—the youngest of which slept with him during the early part of his sickness. The swelling and inflammation continued, until it involved nearly the whole side of the face, closing the right eye, etc., when suppuration took place, the abscess pointing under the lobe of the ear, near the angle of the jaw. I then lanced it, evacuating at least two fluid ounces of pus, of a dark green color. I was then in hopes that the abscess would readily heal, and my patient get better ; but in this I was disappointed. The suppuration continued, my patient got worse, with strong symptoms of pyæmia. I became alarmed. I injected the cavity with a solution of carbolic acid and tincture of iodine, as strong as my patient could bear it. Having no other guide for the strength of my solution, I used it mornings and evenings for two days, when a portion of one of the lobes of the gland made its appearance at the opening of the abscess. I managed to remove it with my forceps and scissors, cutting it away in pieces, as best I could. Following up the injections, I managed in the course of three or four days to remove the entire gland in the same manner. It left a free, open cavity, the opening being the size of a ten cent piece. Looking down upon the carotid artery at its bi-furcation, displaying at full view the external and internal branches, the sight was truly beautiful. The dissection was complete, nature having done her work as no scalpel in the hands of man could have laid bare this delicate region in such a manner. The silvery-coated artery, for the space of an inch and a half, had been stripped of its surrounding tissues, and lay there, seemingly unharmed, sending the life-giving fluid along in its continued circulation. From this time he readily convalesced, only losing the use of the facial nerve, which produced an awkward appearance of the face when laughing. His treatment was tincture of iron and quinine, given internally, all that the system would tolerate.

Case II. — Peshtigo, Wisconsin, August 16th, 1872 : Called to see a patient of Dr. G. R. Bartram, who had given him up to die—Joseph Martin, aged 19; Irish; a native of Canada; laboring under typho-remittent fever; at the end of the third week of the disease, presenting the following conditions : Patient much emaciated; skin hot and dry; pulse 90 per minute; hurried breathing; lips thin and retracted, dry and parched; sordes upon the teeth; tongue coated with a dark brown fur; edges and tip red, but still maintained a little moisture; abdomen slightly tympanitic, with diarrhœa, having from four to twelve evacuations during the twenty-four hours; countenance anxious; the mind wandering and delirious at times, with considerable deafness; both parotid glands badly swollen, red and painful, with marked symptoms of suppuration. The inflammation was supposed to be erysipelatous, by Dr. Bartram, who said that it had commenced upon the side of the nose, and spread to the glands three days before. As he had been taking largely of tincture of iron and the sulphite of soda, I concluded that it might be irritating the alimentary canal too much; so I gave for the diarrhœa the following, in teaspoonful doses, every four hours: Sulph. magnesia, ʒ ii; Tinct. opii, ʒ ii; arom. sulph. acid, ʒ i; mint water, ℥ ii.

And for the object of maintaining the tonicity of the nervous system, as well as the functions of assimilation and conversion of food into good, healthy tissue, thereby assisting in the elimination of the morbific matters already circulating in the blood, I gave the following, in teaspoonful doses, every six hours, alternated with two grains of quinine: Nitric acid, ʒ i; strychnia, gr. ii; simple syrup and water, aa ℥ ii.

August 17th.—Patient more rational; takes more nutriment, chiefly milk; pulse 80 per minute; diarrhœa checked; parotids still badly swollen, and painful; very deaf, with a thin, bloody discharge from both ears; inject and wash them out with warm soap suds; also bathe the upper and lower extremities; quinine and strychnine solution continued.

August 19th, 10 A. M.—Patient complains of great pain in the jaws and ears; had a restless night; discharges the same; abscess pointed under the lobes of both ears; lance them, and evacuate about two ounces of dark green-colored pus from each abscess, and inject the cavities with a strong solution of carbolic acid and water, the injections passing readily out through the ears, thus showing a direct opening from the abscess through the external canals of both of them. This had the effect to relieve the pain and fever somewhat. Saw him again in the afternoon. The discharge was very great. After removing all of the pus that I could, I discovered small shreds of the gland appearing at the opening of the abscesses. By careful manipulation, with a small pair of forceps and scissors, I was able to remove several loose portions that had sloughed from the position. I injected the cavities as before, using the solution as strong as he could bear it, and placed him upon a diet of corn meal gruel.

August 20th.—Patient improving; still considerable discharge of pus; succeeded in removing several pieces of gland, from half an inch to an inch and a half in length—one section was two inches long, and weighed nearly a drachm.

August 21st.—Patient still improving; removed the balance of gland tissue, leaving a free, open cavity, similar to Case I.; inject as usual. From this time forward he made a rapid and complete recovery, having the full use of the facial nerves.

*Remarks.*—In the above cases it is plain that suppuration of the parotid glands took place, and apparently from entire different causes, and under entire different circumstances. In both cases the patients were reduced to the lowest condition of vitality consistent with their constitutions; yet, on the removal of the gland, they rapidly recovered.

Question.—Is there any known means to prevent the suppuration of the gland, under the above circumstances, and save the life of the patient? If not, does not the salvation of the patient depend upon the early removal of the gland?

